# Supplementary material for: High frequency neural spiking and auditory signaling by ultrafast red-shifted optogenetics
Source: Nat Commun. 2018 May 1;9:1750. doi: 10.1038/s41467-018-04146-3 (PMC5931537; doi:10.1038/s41467-018-04146-3)
Supplement: Supplementary file 1 — Supplementary Information [file 41467_2018_4146_MOESM1_ESM.docx]

**High frequency neural spiking and auditory signaling by ultrafast red-shifted optogenetics**

Mager *et al*.


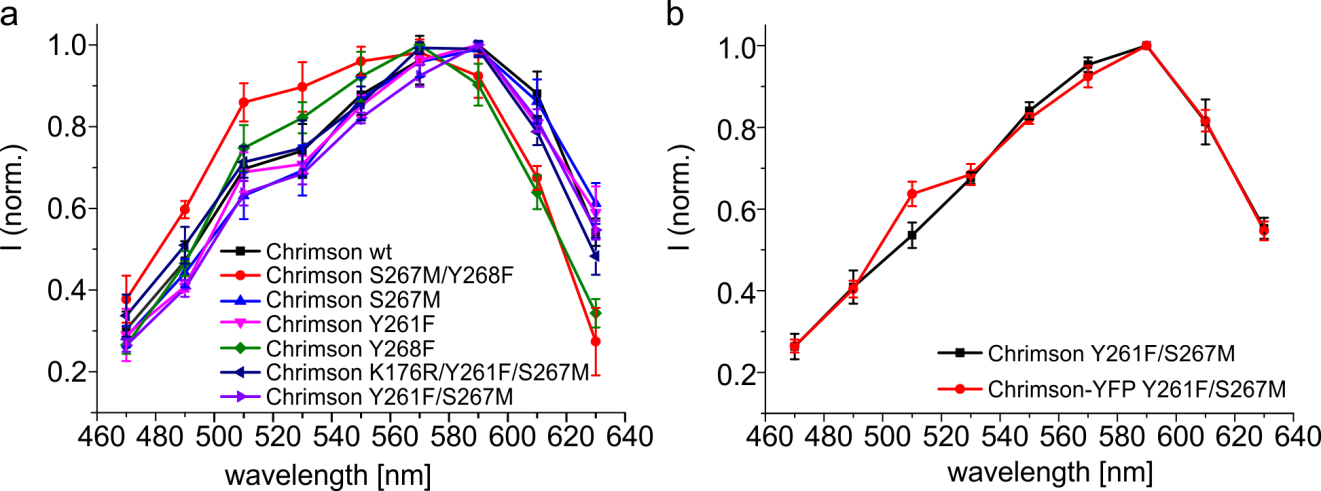


**Supplementary Figure 1.** Action spectra of Chrimson and Chrimson mutants. Shown are normed peak currents in response to ns light-pulses of indicated wavelength. The electrophysiological recordings were performed in NG cells by patch-clamp experiments in the whole-cell configuration at a membrane potential of -60 mV. **(a)** Action spectra of Chrimson-EYFP (
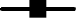
, n=6), Chrimson-EYFP S267M/Y268F (
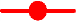
, n=3), Chrimson-EYFP S267M (
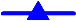
, n=3), Chrimson-EYFP Y261F (
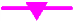
, n=4), Chrimson-EYFP Y268F (
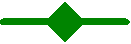
, n=5), Chrimson-EYFP K176R/Y261F/S267M (vf-Chrimson-EYFP) (
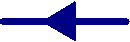
, n=4) and Chrimson-EYFP Y261F/S267M (f-Chrimson-EYFP) (
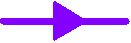
, n=4). **(b)** Action spectra of Chrimson Y261F/S267M (f-Chrimson) (
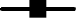
, n=3) and Chrimson-EYFP Y261F/S267M (f-Chrimson-EYFP) (
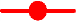
, n=4). The pulse energies at the different wavelengths were set to equal photon counts for the recordings of the action spectra. Experimental details are described in the Methods section.

**
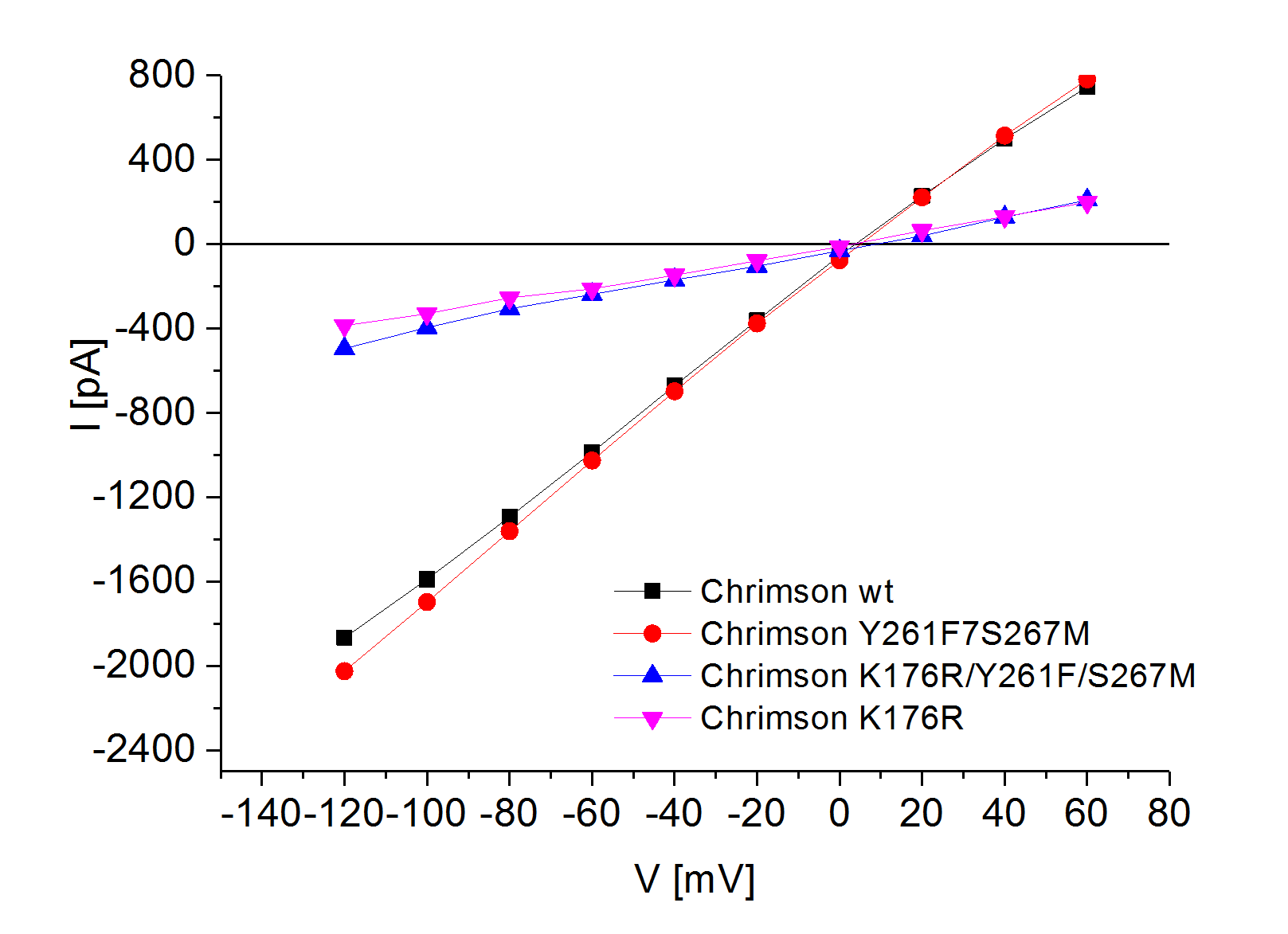
**

**Supplementary Figure 2.** Current (I) - voltage (V) curves of Chrimson and Chrimson mutants. Shown are typical IV curves of Chrimon-EYFP wt, Chrimson-EYFP Y261F/S267M (f-Chrimson-EYFP), Chrimson-EYFP K176R/Y261F/S267M (vf-Chrimson-EYFP) and Chrimson-EYFP K176R recorded in NG cells. Whole-cell patch-clamp experiments were performed under voltage-clamp conditions. The photocurrents were measured in response to 3 ms light-pulses with a wavelength of 594 nm and a saturating light intensity of 23 mW/mm^2^. Peak currents were taken for the quantification.

**
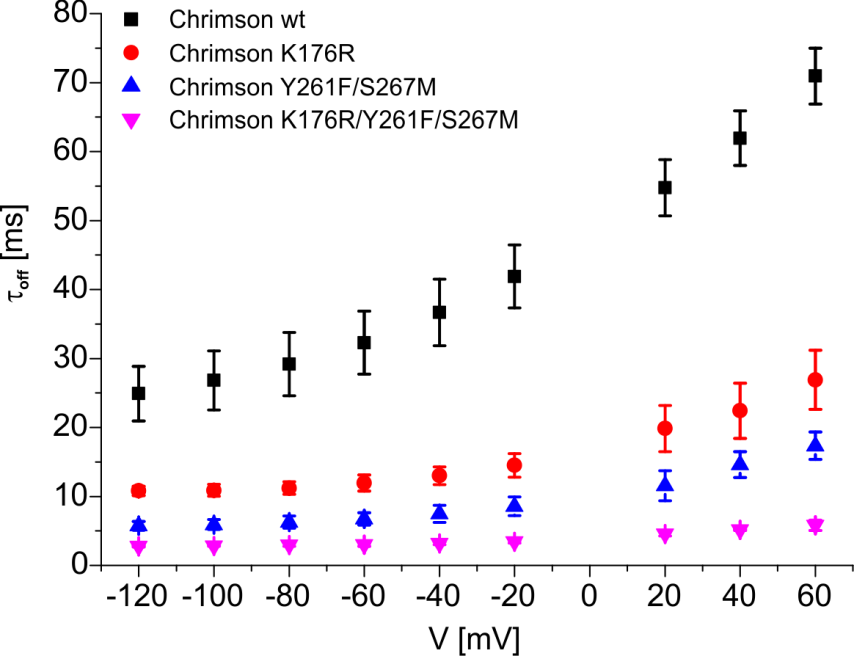
**

**Supplementary Figure 3.** Off-kinetics of Chrimson and Chrimson mutants at different membrane potentials. Whole-cell patch-clamp experiments were performed under voltage-clamp conditions. Photocurrents were measured in response to 3 ms light pulses with a saturating intensity of 23 mW/mm^2^ and a wavelength of 594 nm. Shown is the average τ_off_ for Chrimson-EYFP (
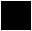
, n=3), Chrimson-EYFP K176R (
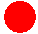
, n=4), Chrimson-EYFP Y261F/S267M (f-Chrimson-EYFP) (
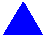
, n=3) and Chrimson-EYFP K176R/Y261F/S267M (vf-Chrimson-EYFP) (
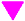
, n=3). The error bars represent the standard deviation. The τ_off_ values were determined as described in the Methods section.

**
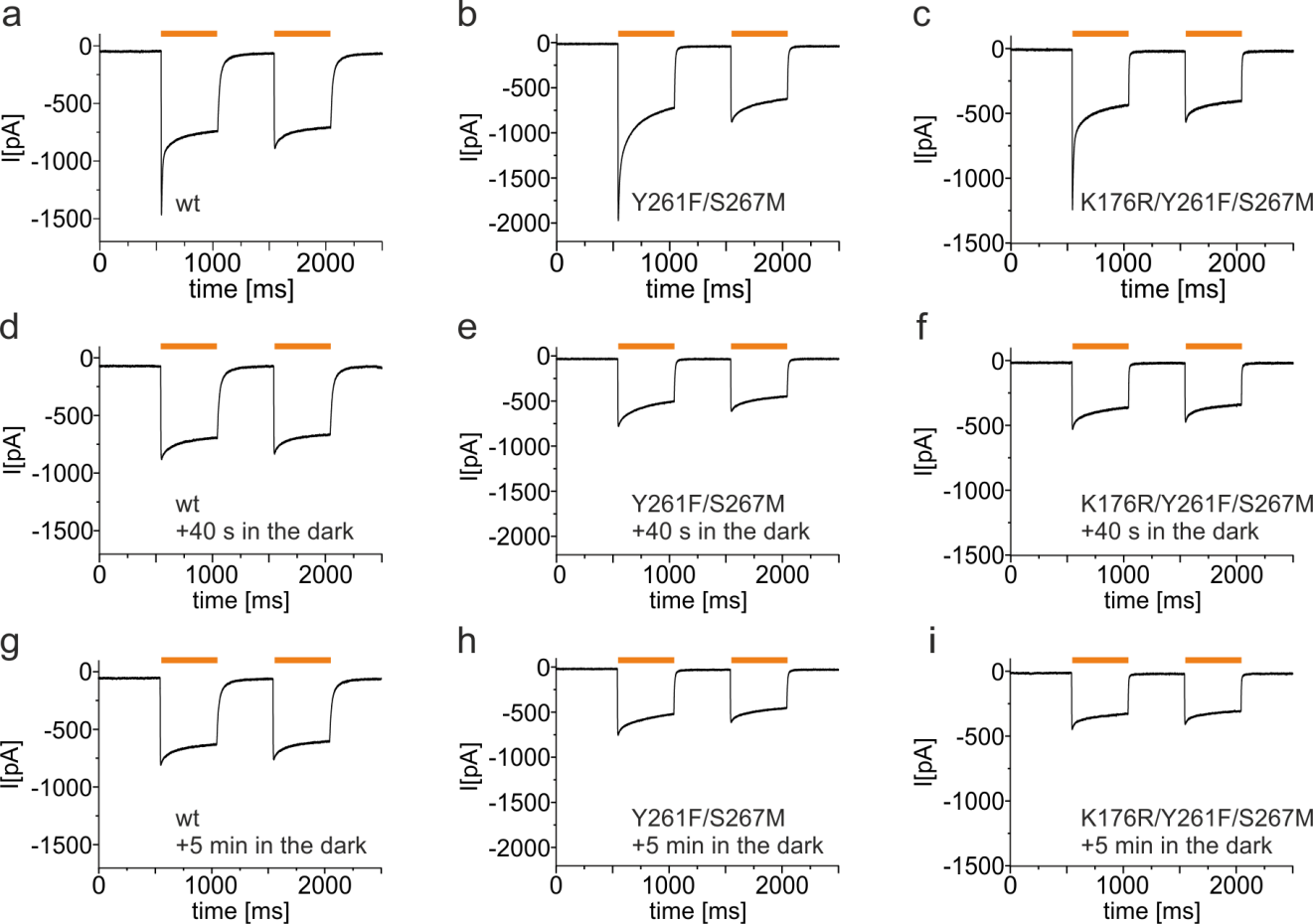
**

**Supplementary Figure 4.** Peak current inactivation and recovery of Chrimson and Chrimson mutants. Typical photocurrents in response to a double illumination protocol using 500 ms light pulses with a saturating intensity of 23 mW/mm^2^ and a wavelength of 594 nm. The photocurrents of Chrimson-EYFP **(a,d,g)**, Chrimson-EYFP Y261F/S267M (f-Chrimson-EYFP) **(b,e,h)** and Chrimson-EYFP K176R/Y261F/S267M (vf-Chrimson-EYFP) **(c,f,i)** were measured by patch-clamp experiments in the whole-cell configuration at a membrane potential of -60 mV. The double illumination protocol was repeated after a waiting time of 40 s **(d,e,f)** and 5 min **(g,h,i)** in the dark.

**
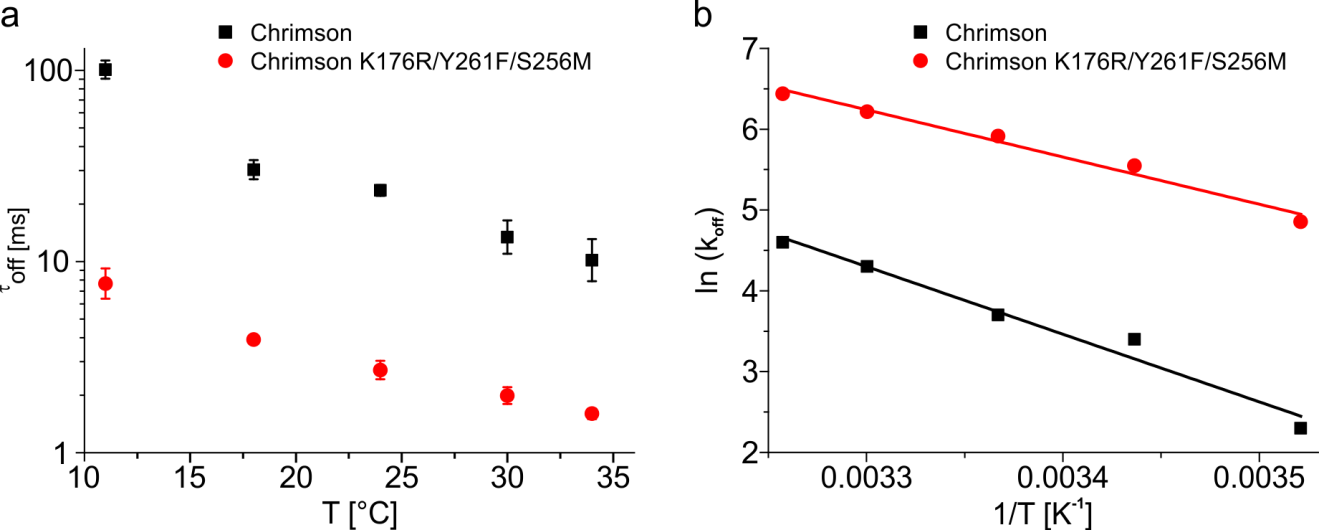
**

**Supplementary Figure 5.** Temperature dependence of the off-kinetics of Chrimson and Chrimson K176R/Y261F/S267M (vf-Chrimson). **(a)** Shown are the average τ_off_ values (n=3-7) and the corresponding standard deviations. NG cells transiently expressing Chrimson-EYFP and Chrimson-EYFP K176R/Y261F/S267M were investigated by patch-clamp measurements in the whole-cell configuration at a membrane potential of -60 mV. All photocurrents, but the photocurrents recorded at 34°C, were measured in response to 3 ms light pulses with an intensity of 23 mW/mm^2^ and a wavelength of 594 nm. The photocurrents recorded at a temperature of 34°C were measured in response to light-pulses with a pulse length of 7 ns and a wavelength of 594 nm, which were generated with the Opolette 355 tunable laser system (Opotek Inc). Thereby the pulse energy was set to value of 10^19^ photons/m^2^. The τ_off_ values were determined by a fit of the decaying photocurrents to a monoexponential function. **(b)** Arrhenius plot of the data shown in A. The lines indicate activation energies of 69.6 kJ/mol for Chrimson and 48.5 kJ/mol for Chrimson K176R/Y261F/S267M.

**
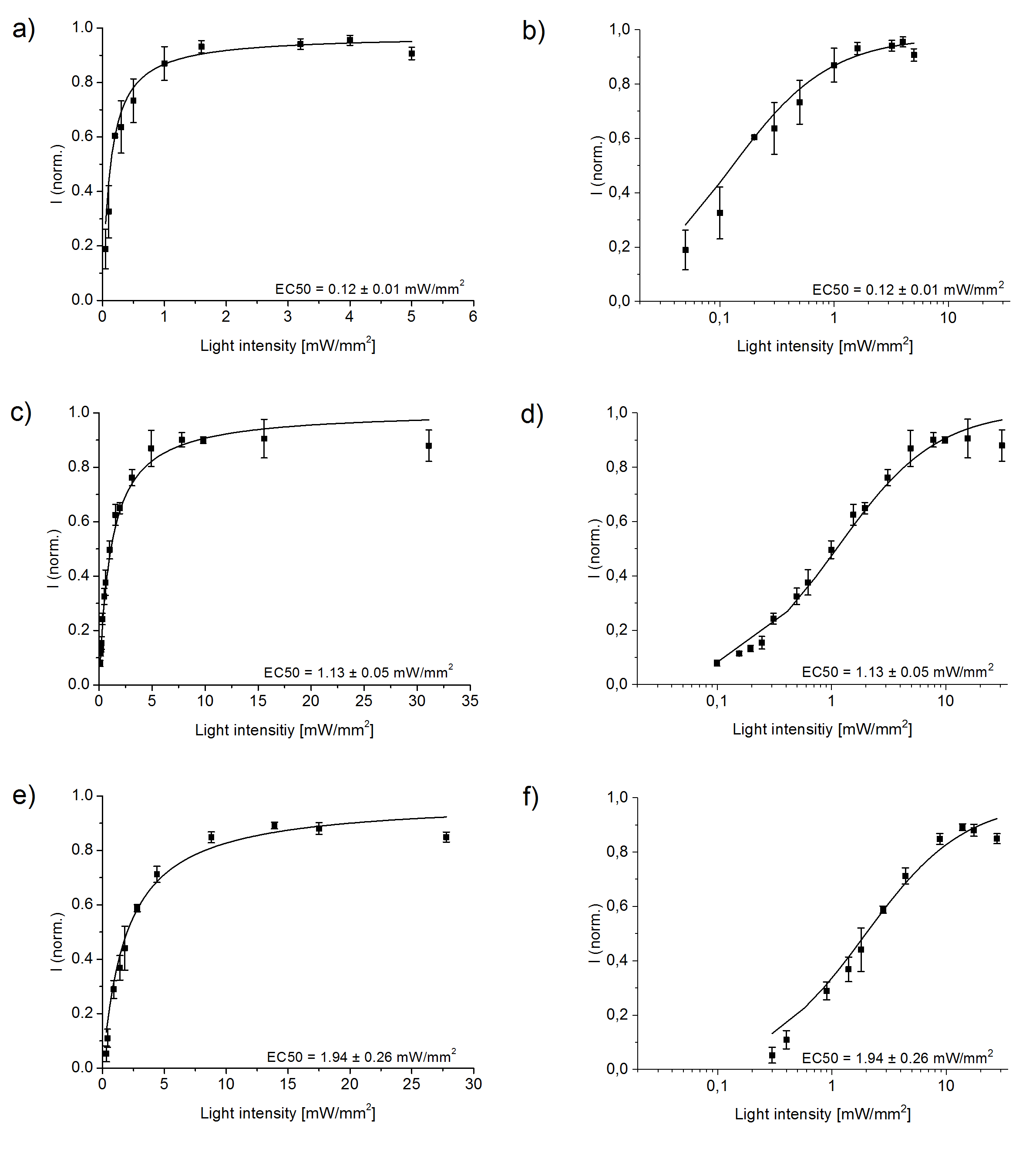
**

**Supplementary Figure 6.** Light intensity dependence of Chrimson and Chrimson mutants. Chrimson-EYFP **(a,b)**, f-Chrimson-EYFP **(c,d)** and vf-Chrimson-EYFP **(e,f)** were investigated in NG108-15 cells by patch-clamp experiments in the whole-cell configuration at a membrane potential of -60 mV. The photocurrents were measured in response to 500 ms light-pulses of indicated light intensities (λ=594 nm). The graphs show average values from normed stationary photocurrents (n=3) and the corresponding standard deviations. The solid line is a fit to the data using a hyperbolic model function with a half-saturating value EC50 and a fixed v_max_=1. The light intensity is shown in linear scale (**a,c,e**) and in logarithmic scale (**b,d,f**).

**
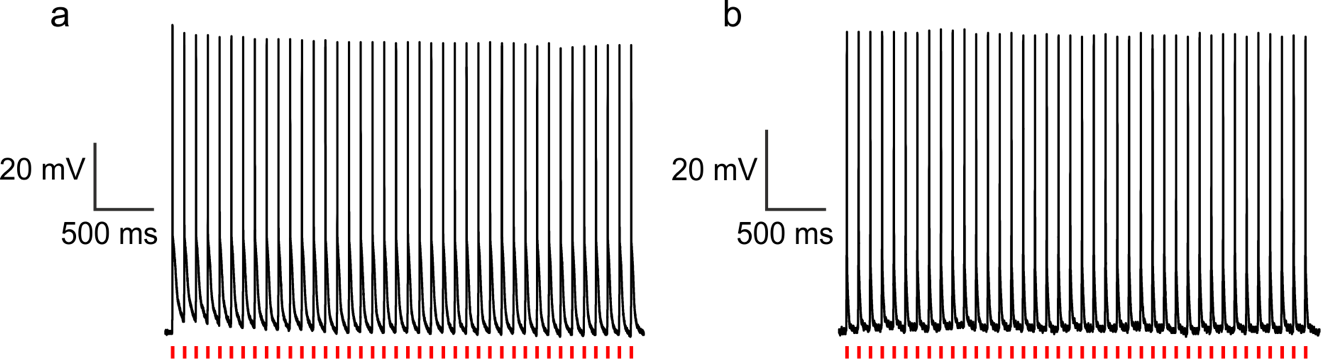
**

**Supplementary Figure 7.** Red light triggered spiking in rat hippocampal neurons. Rat hippocampal neurons heterologously expressing f-Chrimson-EYFP **(a)** and vf-Chrimson-EYFP **(b)** were investigated by patch-clamp experiments in the whole-cell configuration under current-clamp conditions. The action potentials were triggered by 40 light-pulses (ν = 10 Hz) with a pulse width of 3 ms, a wavelength of 640 nm and a saturating light intensity of 23 mW/mm^2^.

**
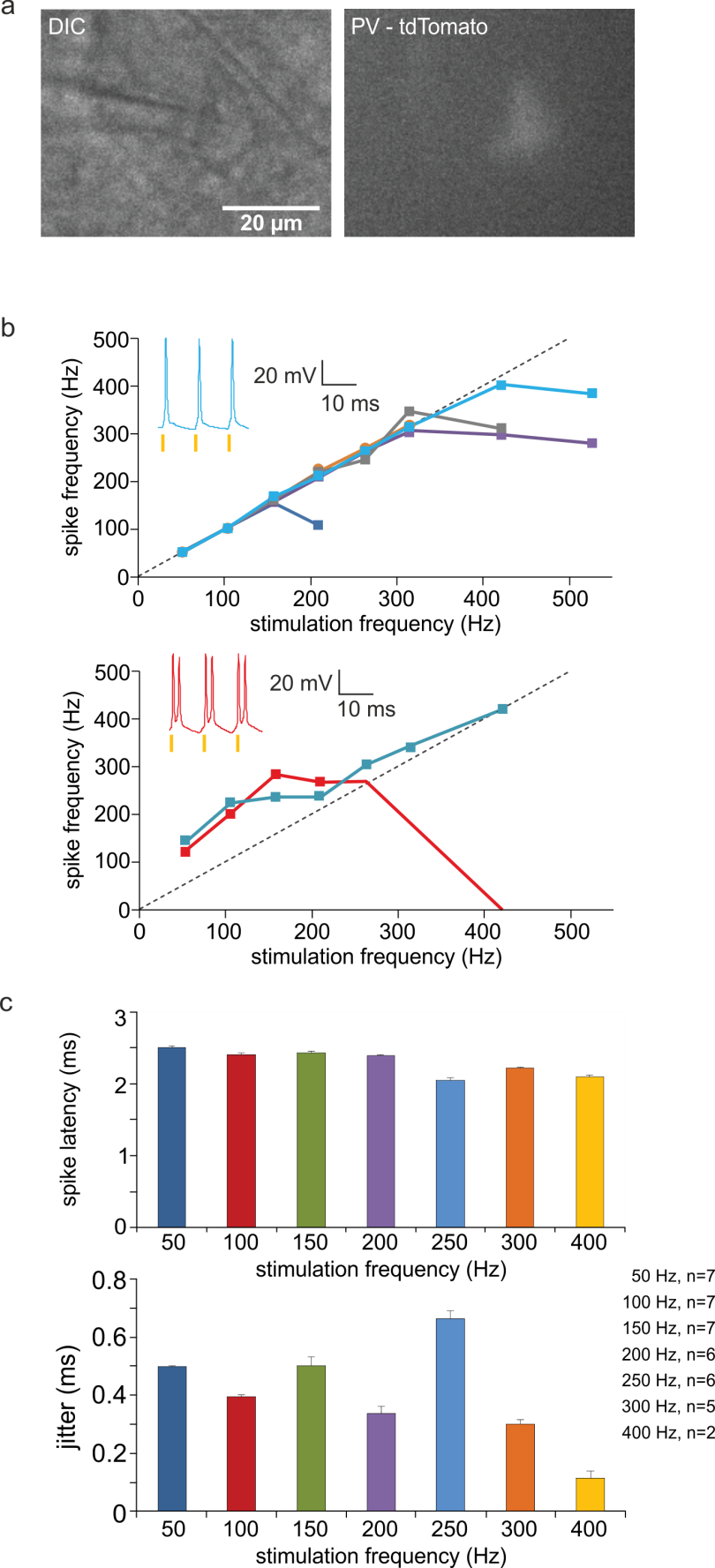
**

**Supplementary Figure 8.** Fidelity and temporal precision of light triggered spiking in parvalbumin-positive interneurons heterologously expressing vf-Chrimson-EYFP. **(a)** Example IR-DIC and fluorescent image of a recorded neocortical parvalbumin-positive interneuron identified by red fluorescence in an acute slice. **(b)** Action potential frequency as a function of stimulation frequency. Most neurons followed optical stimulation faithfully with 1 action potential / light pulse up to a maximum, and then plateaued off (top, n=5). In 2 interneurons, single light pulses could also elicit action potential doublets (bottom, n=2). We note that optimization of expression time and stimulation light density is expected to minimize this caveat. Insets: example traces of cells shown in blue and red at 100 Hz stimulation frequency. **(c)** Upper panel: action potential latency (assessed at peak) after light pulse onset for all stimulation frequencies with reliable spiking (> 85%). (non-parametric Friedman test with a post hoc Dunn’s multiple comparison test revealed the following significant differences: 50HZ vs 250Hz, ***; 50HZ vs 300Hz, ***; 100Hz vs 250Hz, ***; 100Hz vs 300Hz *; 150Hz vs 250Hz, ***; 150Hz vs 300Hz, **; 200Hz vs 250Hz, ***.) Lower panel: action potential jitter (s.d. of latencies) for the different stimulation frequencies (average of 20 spikes/trace and 10 traces). (non-parametric Friedman test with post hoc Dunn’s multiple comparison test revealed the following significant differences: 50HZ vs 200Hz, ***; 50HZ vs 300Hz, ***; 100Hz vs 250Hz, ***; 150Hz vs 200Hz, *; 150Hz vs 300Hz, ***; 200Hz vs 250Hz, ***; 250Hz vs 300Hz, ***.). Error bars are s.e.m; p ≤ 0.05 = *, p ≤ 0.01 = **, p ≤ 0.001 = ***.

**
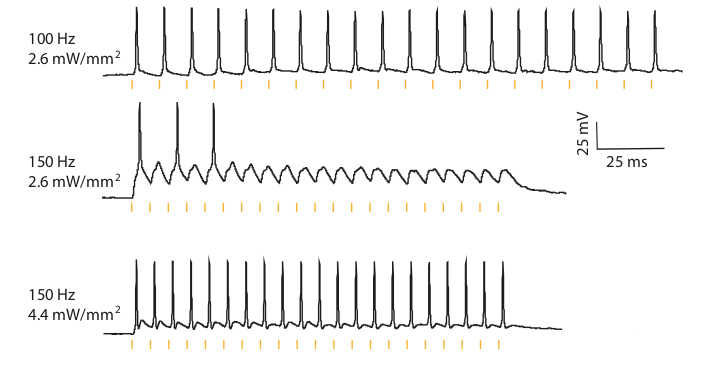
**

**Supplementary Figure 9.** Precise optogenetic control of parvalbumin-positive interneurons depends on irradiance and stimulation frequency. Exemplary traces showing precise spiking at 100 Hz stimulation frequency, but stimulation failures at 150 Hz for the same irradiance level (2.6 mW/mm^2^). Increasing the light intensity restored precise spiking (bottom).


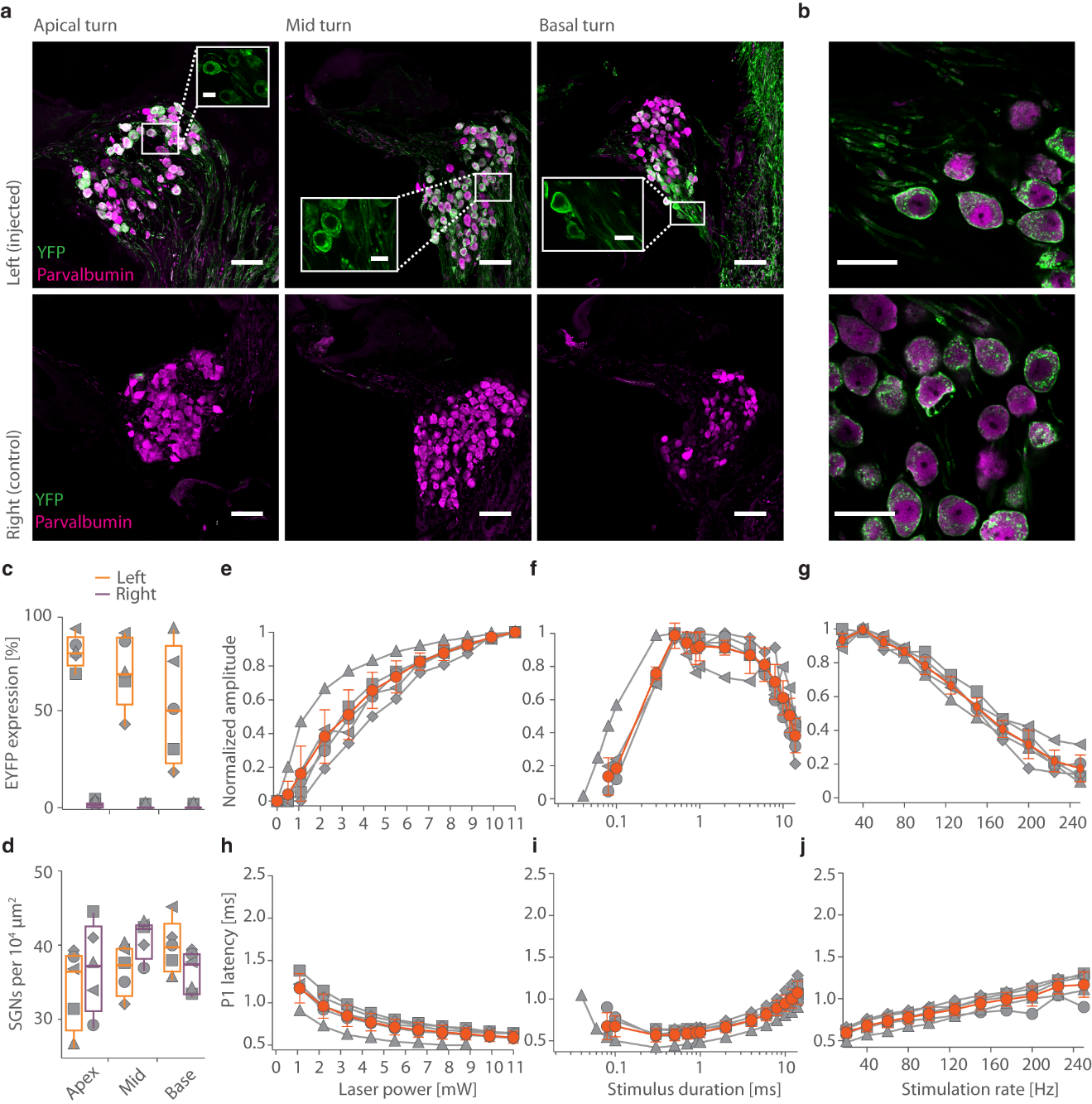


**Supplementary Figure 10.**  f-Chrimson expression and function in SGNs in aged Bl6/J mice. **(a)** Projections of confocal cryosections (scale bar: 50µm) with EYFP (green) and parvalbumin (magenta) immunofluorescence of SGNs in three cochlear regions. Insets (scale bar: 10µm) show close-up images of single z-sections of the same images. **(b)** Confocal z-sections (0.25 µm step) showing EYFP expression (green) in parvalbumin positive SGNs (magenta) from cochlear cryosections of the injected ear of the animal shown in **a**. Scale bar: 20 µm. **(c)** Fraction of EYFP-positive SGNs (identified by parvalbumin immunofluorescence, parvalbumin^+^) and **(d)** density of parvalbumin^+^ SGNs (#cells per 10^4^ µm^2^) obtained from data as in **Fig. 4f** for apex, mid-cochlea (Mid) and base of the cochlea. Symbols mark results from individual animals (also for **e-j**, *n =* 5) and box plots show 10^th^, 25^th^, 50^th^, 75^th^ and 90^th^ percentiles of the injected (orange) and control (magenta) cochleae (one-way ANOVA, *P* = 0.1646; F_2,12_ = 2.1; post-hoc Tukey’s test for comparison of expression, *P* > 0.05 for all pairwise comparisons; t-test for comparison of density, L_apex_ vs R_apex_, L_mid_ vs R_mid_, L_base_ vs R_base_  *P*  > 0.05 for all comparisons). **(e-g)** Normalized P1-N1-amplitude as a function of laser intensity (**e,** 1 ms at 20 Hz), pulse duration (**f**, 11 mW at 20 Hz), and stimulus rate **(g,** 11 mW, 1 ms). Group average (lines) and s.d. (error bars) are shown in orange (same for g-i). **(h-j)** P1-latency as a function of laser intensity (**h**, as in **e**), duration (**i**, as in **f**), and rate (**j**, as in **g**).

**Supplementary Figure 11.** Latency and temporal jitter of f-Chrimson-mediated spiking in SGNs. **(a)** Spike latency to light pulse onset as a function of stimulus rate (1 ms pulses of 11 mW over 400 or 900 ms stimulation, leaving 100 ms of inter-train recovery, only first 400 ms were analyzed here). Mean estimates were obtained for trials containing in average 5 spikes or more for each neuron. **(b)** Spike latency considering three time-windows from light pulse-train onset. **(c)** Same analysis as in **(b)** for temporal jitter of spikes, showing that this parameter tends to increase at later time windows along the train, which reflects a decrease in temporal precision of spiking. Data points show mean ± s.e.m. (error bars). Number of units (from 5 mice) included for each stimulation frequency (color coded) is shown in **a.**


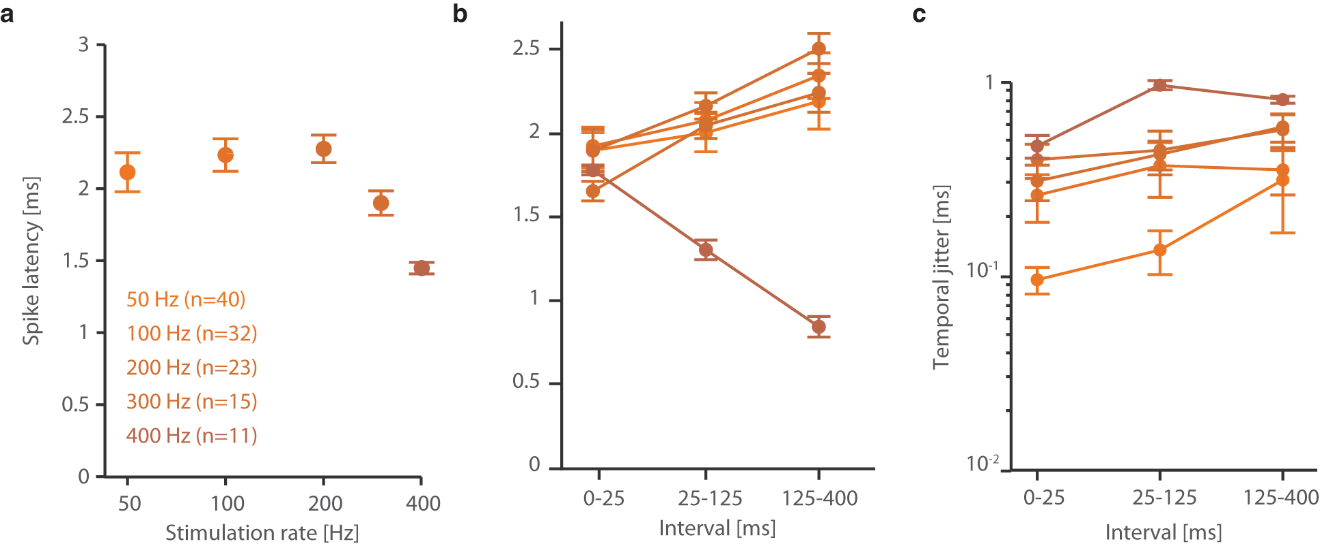


**Supplementary Table 1. Relative calcium permeabilities of channelrhodopsin variants.**

**
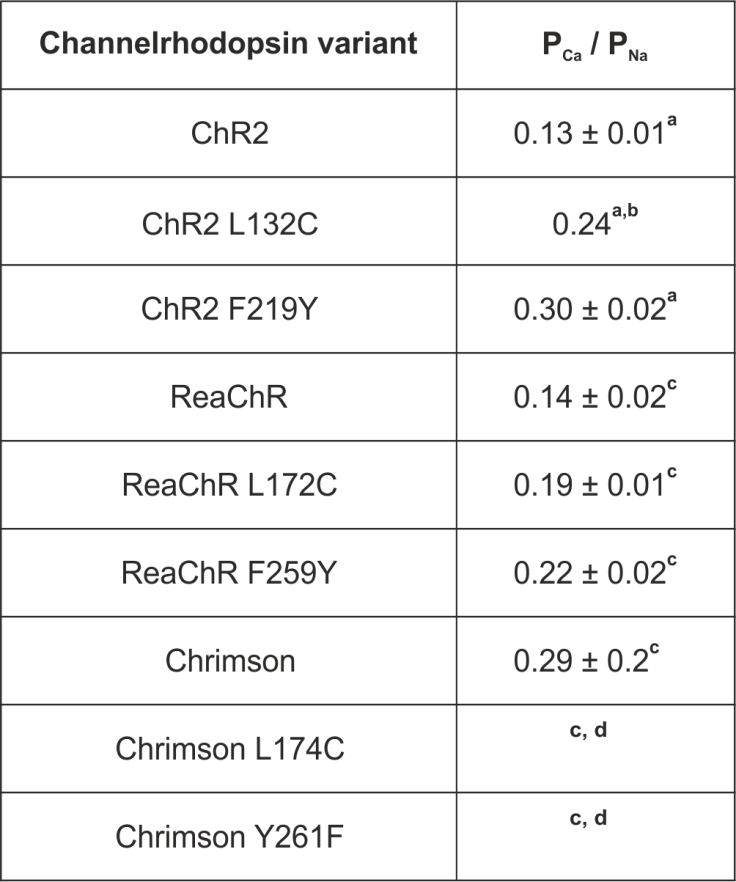
**

Shown are the average, relative calcium permeabilities (P_Ca_/P_Na_, n=3-5) and the corresponding standard deviations. The values were determined in NG cells and HEK cells as described in the Methods section. ^a^ The relative calcium permeabilities were determined in HEK cells. ^b^ taken from[^10^](#_ENREF_1) ^c^ The relative calcium permeabilities were determined in NG cells. ^d^ Determination of relative calcium permeability not possible due to strongly reduced calcium currents.

**Supplementary Table 2. Current densities of Chrimson and Chrimson mutants**

| **Chrimson variant** | **J_-60 mV_ [pA/pF]** |
| --- | --- |
| wt ^§^ | 24.0 ± 6.8 ^# b*,d***, f*, h***, i***, j*, k***^ |
| K176R ^$^ | 10.1 ± 6.9 ^# a*, e***, g***^ |
| S267M ^§§^ | 22.6 ± 13.3 ^# d***, h**, i***, j*, k***^ |
| Y268F ^$^ | 3.5 ± 1.6 ^# a***, c***, e***, g***^ |
| Y261F ^§^ | 33.3 ± 8.6 ^# b***, d***, f***, h***, i***, j***, k***^ |
| S267M/Y268F ^$^ | 10.8 ± 5.9 ^# a*, e***, g***^ |
| Y261F/S267M ^§^ | 34.2 ± 12.7 ^# b***, d***, f***, h***, i***, j***, k***^ |
| K176R/S267M/Y268F ^$^ | 4.7 ± 2.7 ^# a***, c**, e***, g***^ |
| Y261F/S267M/Y268F ^$^ | 6.0 ± 4.7 ^# a***, c***, e***, g***^ |
| K176R/Y261F/S267M ^$^ | 8.3 ± 5.3 ^# a*, c*, e***, g***^ |
| K176R/Y261F/S267M/Y268F ^$^ | 2.6 ± 0.9 ^# a***, c***, e***, g***^ |

Current densities (J_-60mV_) of Chrimson and Chrimson mutants. Shown are the average current densities (n=7-11) and the corresponding standard deviations. NG cells transiently expressing Chrimson-EYFP and Chrimson-EYFP mutants were investigated by patch-clamp measurements in the whole-cell configuration at a membrane potential of -60 mV. The J_-60mV_ values were determined as described in the Methods section. ^#^ Significantly different current density compared to Chrimson a) wt b) K176R c) S267M d) Y268F e) Y261F f) S267M/Y268F g) Y261F/S267M h) K176R/S267M/Y268F i) Y261F/S267M/Y268F j) K176R/Y261F/S267M k) K176R/Y261F/S267M/Y268F (ANOVA with Bonferroni post-hoc test: * p < 0.05 ** p < 0.01 *** p < 0.001) ^§^ variants with higher current density (no significantly different current densities within the group) ^$^ mutants with lower current density (no significantly different current densities within the group) ^§§^ no significantly different current density compared to the high current density group and significantly different current density compared to all members of the low current density group, but Chrimson K176R and Chrimson S267M/Y268F

**Supplementary Table 3. Relative potassium and proton permeabilities of Chrimson variants.**

**
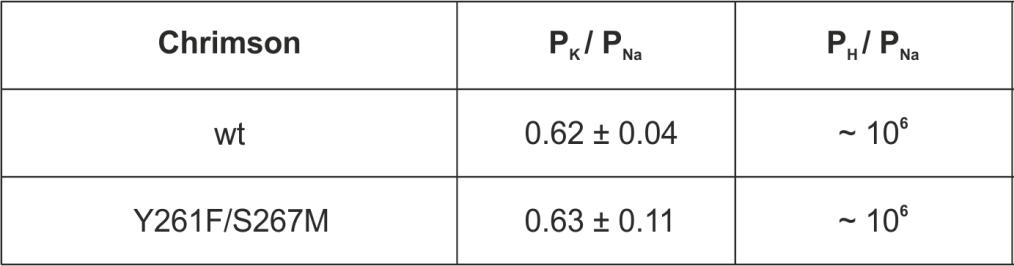
**

Shown are the average, relative potassium permeabilities (P_K_/P_Na_, n=5) and the average, relative proton permeabilities (P_H_/P_Na_, n=7) of Chrimson-EYFP and Chrimson-EYFP Y261F/S267M. The values were determined in Xenopus leavis oocytes as described in the Methods section.

**Supplementary Table 4. Off kinetics (τ_off_), current density (J_-70mV_) and the lowest light intensity required to induce action potentials with a probability of 100 % (I_100_).**

Shown are the average τ_off_ (n=3-11), the average J_-70mV_ (n=10-14), the average I_100_ (n=15) and the corresponding standard deviations. The τ_off_, J_-70mV_ and I_100_ values were determined in rat hippocampal neurons as described in the Methods section.

**Supplementary Table 5. List of forward primers used for site-directed mutagenesis**

| Channelrhodopsin variant | forward primers |
| --- | --- |
| ChR2 F219Y | GTGACTGGCATGGCTTGGCTGTACTTCGTGTCCTGGGGGATG |
| ReaChR F259Y | ATGGCCTGGCTGTACTTCGTGAGCTGGGGCATGTTC |
| ReaChR L172C | GCTGCTGACCTGCCCCGTGATTTGCATTCATCTGAGCAACCTGACCGGCC |
| VChR1 F214Y | GTGCGGGTGATGGCATGGACCTACTTTGTGGCCTGGGGGATGTTC |
| VChR1 L127C | CTGCTGACCTGTCCCGTCCTGTGCATTCATCTGTCCAATCTGACC |
| Chrimson Y261F | GTCGTGAAGCTGATGGCCTGGGCTTACTTCGCCTCTTGG |
| Chrimson L174C | CTGCTGTCTTGCCCCGTGATCTGCATCAAGCTGAGCAACCTGAGC |
| Chrimson S267M | TACTTCGCCTCTTGGGGCATGTACCCAATCCTCTGGGCAGTG |
| Chrimson Y268F | TTCGCCTCTTGGGGCAGCTTCCCAATCCTCTGGGCAGTG |
| Chrimson K176R | CCCGTGATCCTGATCCGACTGAGCAACCTGAGC |
| Chrimson S267M/Y268F | TACTTCGCCTCTTGGGGCATGTTCCCAATCCTCTGGGCAGTG |
| Chrimson Y261F/S267M | GTCGTGAAGCTGATGGCCTGGGCTTACTTCGCCTCTTGG |
|  | TACTTCGCCTCTTGGGGCATGTACCCAATCCTCTGGGCAGTG |
| Chrimson K176R/S267M/Y268F | TACTTCGCCTCTTGGGGCATGTTCCCAATCCTCTGGGCAGTG |
|  | CCCGTGATCCTGATCCGACTGAGCAACCTGAGC |
| Chrimson Y261F/S267M/Y268F | TACTTCGCCTCTTGGGGCATGTTCCCAATCCTCTGGGCAGTG |
|  | GTCGTGAAGCTGATGGCCTGGGCTTACTTCGCCTCTTGG |
| Chrimson K176R/Y261F/S267M | GTCGTGAAGCTGATGGCCTGGGCTTACTTCGCCTCTTGG |
|  | TACTTCGCCTCTTGGGGCATGTACCCAATCCTCTGGGCAGTG |
|  | CCCGTGATCCTGATCCGACTGAGCAACCTGAGC |
| Chrimson K176R/Y261F/S267M/Y268F | GTCGTGAAGCTGATGGCCTGGGCTTACTTCGCCTCTTGG |
|  | TACTTCGCCTCTTGGGGCATGTTCCCAATCCTCTGGGCAGTG |
|  | CCCGTGATCCTGATCCGACTGAGCAACCTGAGC |

**Supplementary Table 6. List of reverse primers used for site-directed mutagenesis**

| Channelrhodopsin variant | Reverse primers |
| --- | --- |
| ChR2 F219Y | CAT CCC CCA GGA CAC GAA GTA CAG CCA AGC CAT GCC AGT CAC |
| ReaChR F259Y | GAACATGCCCCAGCTCACGAAGTACAGCCAGGCCAT |
| ReaChR L172C | GGCCGGTCAGGTTGCTCAGATGAATGCAAATCACGGGGCAGGTCAGCAGC |
| VChR1 F214Y | GAACATCCCCCAGGCCACAAAGTAGGTCCATGCCATCACCCGCAC |
| VChR1 L127C | GGTCAGATTGGACAGATGAATGCACAGGACGGGACAGGTCAGCAG |
| Chrimson Y261F | CCAAGAGGCGAAGTAAGCCCAGGCCATCAGCTTCACGAC |
| Chrimson L174C | GCTCAGGTTGCTCAGCTTGATGCAGATCACGGGGCAAGACAGCAG |
| Chrimson S267M | CACTGCCCAGAGGATTGGGTACATGCCCCAAGAGGCGAAGTA |
| Chrimson Y268F | CACTGCCCAGAGGATTGGGAAGCTGCCCCAAGAGGCGAA |
| Chrimson K176R | GCTCAGGTTGCTCAGTCGGATCAGGATCACGGG |
| Chrimson S267M/Y268F | CACTGCCCAGAGGATTGGGAACATGCCCCAAGAGGCGAAGTA |
| Chrimson Y261F/S267M | CACTGCCCAGAGGATTGGGAACATGCCCCAAGAGGCGAAGTA |
|  | GCTCAGGTTGCTCAGTCGGATCAGGATCACGGG |
| Chrimson K176R/S267M/Y268F | CCAAGAGGCGAAGTAAGCCCAGGCCATCAGCTTCACGAC |
|  | CACTGCCCAGAGGATTGGGTACATGCCCCAAGAGGCGAAGTA |
| Chrimson Y261F/S267M/Y268F | CACTGCCCAGAGGATTGGGAACATGCCCCAAGAGGCGAAGTA |
|  | CCAAGAGGCGAAGTAAGCCCAGGCCATCAGCTTCACGAC |
| Chrimson K176R/Y261F/S267M | CCAAGAGGCGAAGTAAGCCCAGGCCATCAGCTTCACGAC |
|  | CACTGCCCAGAGGATTGGGTACATGCCCCAAGAGGCGAAGTA |
|  | GCTCAGGTTGCTCAGTCGGATCAGGATCACGGG |
| Chrimson K176R/Y261F/S267M/Y268F | CCAAGAGGCGAAGTAAGCCCAGGCCATCAGCTTCACGAC |
|  | CACTGCCCAGAGGATTGGGAACATGCCCCAAGAGGCGAAGTA |
|  | GCTCAGGTTGCTCAGTCGGATCAGGATCACGGG |
